# Comparative Efficacy of Fermented and Unfermented Ethanolic Rooibos Extracts in Mitigating LPS‐Induced Intestinal Inflammation and Barrier Dysfunction

**DOI:** 10.1002/fsn3.72310

**Published:** 2026-10-30

**Authors:** Shana De Bruyn‐Orr, Mariam Rado, Claudia Abrahams, Stefan Abel, Mariska Lilly

**Affiliations:** ^1^ Applied Microbial and Health Biotechnology Institute Cape Peninsula University of Technology Bellville South Africa; ^2^ Department of Biomedical Science, Faculty of Health and Wellness Science Cape Peninsula University of Technology Bellville South Africa

**Keywords:** *Aspalathus linearis*, barrier protection, ethanolic rooibos extracts, intestinal inflammation, IPEC‐J2, polyphenols

## Abstract

Rooibos ( *Aspalathus linearis* ) is a rich source of bioactive polyphenols; however, most biological studies have focused on traditional aqueous tea extracts, despite extraction solvent and enrichment procedures substantially influencing phytochemical composition and biological activity. This study investigated whether chemically distinct enriched ethanolic extracts prepared from unfermented and fermented rooibos differentially modulate intestinal inflammation and barrier integrity. Following ethanol extraction and Amberlite XAD‐4 enrichment, the extracts were characterized for soluble solids, flavonoid composition and antioxidant capacity before evaluation in an *Escherichia coli* lipopolysaccharide (10 μg/mL)‐stimulated IPEC‐J2 intestinal epithelial cell model. Anti‐inflammatory activity was assessed by determining cell viability, caspase activity, IL‐8 protein production, and gene expression of *IL‐8*, *IL‐6* and *TLR4*, while barrier integrity was evaluated using transepithelial electrical resistance (TEER), ZO‐1 immunofluorescence and tight junction gene expression. Ethanol extraction and enrichment generated extracts with markedly different phytochemical profiles. The enriched unfermented extract contained substantially higher soluble solids (77.72 g/100 g) and aspalathin (60 g/100 g soluble solids) than the fermented extract (53.10 g/100 g and 9.46 g/100 g, respectively), resulting in significantly greater antioxidant activity. Despite these compositional differences, both extracts attenuated LPS‐induced inflammation by reducing IL‐8 protein production and downregulating *IL‐8*, *IL‐6* and *TLR4* gene expression. However, distinct biological activities were observed. The polyphenol‐rich unfermented extract provided superior protection of epithelial barrier integrity, demonstrated by increased TEER, enhanced ZO‐1 localisation and upregulation of tight junction markers, whereas the fermented extract exhibited pronounced anti‐inflammatory activity at lower concentrations despite its lower polyphenol content. These findings demonstrate that post‐harvest processing and extraction technique determine the phytochemical composition of rooibos and consequently influence its dose‐dependent biological activity. Tailoring extraction strategies may therefore enable the development of rooibos extracts towards certain product formulations with targeted anti‐inflammatory or barrier‐protective properties.

## Graphical abstract

Rooibos ( *Aspalathus linearis* ) is rich in bioactive polyphenols, but extraction and processing methods can substantially influence its composition and biological activity. This study investigated enriched ethanolic extracts from unfermented and fermented rooibos in an LPS‐stimulated IPEC‐J2 intestinal epithelial cell model. Despite marked differences in phytochemical composition, both extracts attenuated inflammation. The polyphenol‐rich unfermented extract provided superior epithelial barrier protection, whereas the fermented extract demonstrated pronounced anti‐inflammatory activity at lower concentrations. These findings demonstrate that post‐harvest processing and extraction strategies influence the dose‐dependent biological activity of rooibos and may support the development of targeted anti‐inflammatory or barrier‐protective formulations.

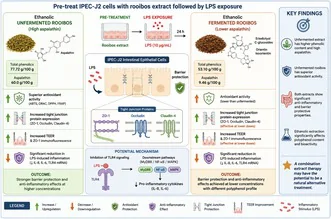

## 1 Introduction

Rooibos ( *Aspalathus linearis* ) is an indigenous South African herbal tea rich in polyphenolic compounds, particularly the unique dihydrochalcones aspalathin and nothofagin, which contribute to its antioxidant and anti‐inflammatory properties (Joubert, Gelderblom, et al. 2008; Afrifa et al. 2023). The therapeutic efficacy of rooibos is, however, not determined solely by its botanical origin but also by the extraction strategy employed. Post‐harvest processing, solvent polarity and extraction conditions substantially influence the recovery, composition, and concentration of phytochemicals, thereby altering the biological activity of the resulting extract. Recent reviews have further highlighted that extraction methodology not only determines polyphenol yield but also preserves compound stability and influences the nutraceutical efficacy of tea‐derived bioactives, making optimisation of extraction conditions essential for developing functional food ingredients and phytotherapeutics (Shaukat et al. 2023).

Although teas are rich in polyphenolic compounds, the recovery and composition of these bioactives are strongly influenced by extraction conditions, particularly solvent type. Solvent polarity determines the solubility and extraction efficiency of individual phytochemicals, thereby affecting the qualitative and quantitative composition of the resulting extract and ultimately its biological activity (Turkmen et al. 2007; Shaukat et al. 2023). Polar solvents, such as water and methanol, are commonly employed for the extraction of polyphenolic compounds (Borges et al. 2020; Jacobsen et al. 2024). Water is traditionally used for tea preparation (Lajoie et al. 2022), however, its efficiency for extracting bioactive compounds is limited (Memon and Idress 2024). In contrast, ethanol, owing to its intermediate polarity, enables the recovery of a broader spectrum of phytochemicals, including flavonoids and phenolic acids, resulting in improved extraction efficiency and potentially enhanced biological activity (Lee et al. 2024; Shaukat et al. 2023). Consequently, the choice of extraction solvent can markedly influence the phytochemical profile, yield and bioactivity of extracts derived from the same plant material.

Although numerous studies have demonstrated anti‐inflammatory properties of aqueous rooibos extracts, comparatively little is known regarding enriched ethanolic extracts and whether altered phytochemical composition translates into different biological activities. In our previous study, aqueous fermented rooibos predominantly attenuated inflammatory responses whereas aqueous unfermented rooibos preferentially preserved epithelial barrier integrity (De Bruyn‐Orr et al. 2025). These findings suggested that differences in phytochemical composition influence biological function. However, it remains unknown whether ethanolic extraction further modifies these biological responses.

The intestinal epithelium serves as a critical barrier between the external environment and the host's internal milieu. The integrity of this barrier is essential for nutrient absorption, immune modulation and defense against pathogens (Zhang et al. 2015; Zang et al. 2026). Disruption of intestinal barrier function and the resulting chronic inflammation are hallmarks of several gastrointestinal disorders, including irritable bowel syndrome (IBS), inflammatory bowel disease (IBD) and colorectal cancer (Bardelčíková et al. 2023), and are frequently associated with oxidative stress and excessive production of pro‐inflammatory cytokines (Choghakhori et al. 2017; Sahoo et al. 2023). Intestinal inflammation is driven by a complex interplay of genetic susceptibility, environmental factors and microbial‐derived compounds, which collectively compromise epithelial barrier integrity and promote chronic immune activation (Pastorelli and Scaldaferri 2018; Shen et al. 2026). Consequently, there is growing interest in natural bioactive compounds that can preserve intestinal barrier function and modulate inflammatory responses. Plant‐derived polyphenols have received considerable attention because of their antioxidant, anti‐inflammatory, antimicrobial, anti‐apoptotic, anti‐diabetic, and anti‐cancer properties (Leri et al. 2020; Stiller et al. 2021; Shaukat et al. 2023). Their anti‐inflammatory activity is largely attributed to the modulation of the nuclear factor kappa B (NF‐κB) signaling pathway, resulting in reduced expression of pro‐inflammatory mediators, including IL‐1β, IL‐6, TNF‐α and CXCL8, through inhibition of IκB degradation or direct suppression of NF‐κB DNA‐binding activity (Karunaweera et al. 2015; Kannan et al. 2025). Among polyphenol‐rich botanical sources, rooibos ( *Aspalathus linearis* ) has emerged as a promising natural therapeutic, with increasing evidence demonstrating its ability to modulate oxidative stress, inflammation and epithelial barrier function.

In vitro intestinal epithelial models are invaluable for investigating the mechanisms underlying inflammation, epithelial barrier function, and the therapeutic potential of bioactive compounds such as polyphenols. The non‐transformed porcine intestinal epithelial cell line (IPEC‐J2) was selected because it is derived from the mid‐jejunum and retains many structural and functional characteristics of normal small intestinal epithelium, including epithelial polarity, tight junction formation and high transepithelial electrical resistance (TEER). IPEC‐J2 closely resembles healthy small intestinal epithelium and expresses key innate immune receptors and inflammatory mediators, including Toll‐like receptors (TLRs), IL‐6 and IL‐8. Consequently, IPEC‐J2 cells are widely used to investigate intestinal barrier function and inflammatory responses to dietary bioactives and other therapeutic compounds (Brosnahan and Brown 2012; Verhoeckx et al. 2015).

Lipopolysaccharide (LPS), a structural component of the outer membrane of Gram‐negative bacteria, is widely used to induce intestinal inflammation in vitro. The lipid A moiety of LPS binds to Toll‐like receptor 4 (TLR4) on intestinal epithelial cells, triggering activation of the NF‐κB signaling pathway and subsequent production of pro‐inflammatory cytokines that contribute to epithelial barrier dysfunction and inflammation (Candelli et al. 2021; Ghosh et al. 2020; Rhee 2014).

Therefore, this study investigated whether ethanolic extraction and enrichment generate chemically distinct unfermented and fermented rooibos extracts with differential biological activities. The extracts were first characterized according to their flavonoid composition and antioxidant capacity before evaluating their anti‐inflammatory and barrier‐protective effects in LPS‐stimulated IPEC‐J2 intestinal epithelial cells. We hypothesized that differences in phytochemical composition resulting from ethanol extraction and polyphenol enrichment would translate into distinct dose‐dependent effects on inflammatory signaling and epithelial barrier integrity. By comparing the biological activities of unfermented (U) and fermented (F) ethanolic rooibos extracts, this study provides mechanistic insights into how extraction method influences the therapeutic potential of rooibos as a natural modulator of intestinal inflammation and barrier dysfunction towards a specific product formulation.

## 2 Materials and Methods

### 2.1 Chemicals

Dulbecco's modified eagles' medium (DMEM) and Ham's F‐12, fetal bovine serum (FBS) and epidermal growth factor (EGF) (Gibco, Thermo Fisher Scientific, Waltham, MA, USA), water‐soluble Dexamethasone (Thermo Fisher Scientific, Waltham, MA, USA), insulin/transferrin/selenium (ITS), Dulbecco's phosphate‐buffered saline (DPBS) and L‐glutamine (L‐Glut) (Capricorn Scientific GmbH, Ebsdorfergrund, Germany), 4‐(2‐hydroxyethyl)‐1‐piperazineethanesulfonic acid (HEPES) (Lonza, Basel, Switzerland), *Escherichia coli* O26:B6 Lipopolysaccharide (LPS), Triton X‐100, dimethyl sulfoxide (DMSO), Amberlite XAD‐4 resin, aspalathin, nothofagin chloroform, methanol, sulfuric acid and p‐anisaldehyde, TLC plates (Sigma‐Aldrich, Merck KGaA, Darmstadt, Germany), ethanol (Kimix, Cape Town, South Africa), bovine serum albumin (BSA) (Biowest, Nuaillé, France).

### 2.2 Tea Extract Preparation and Characterization

#### 2.2.1 Preparation of Tea Extracts

For preparation of the rooibos ethanol extracts, organic sifted unfermented and fermented rooibos tea leaves were obtained from a commercial producer (Carmien Tea, Citrusdal, South Africa). Briefly, a total of 500 g of the respective tea leaves, divided into 100 g batches, were extracted in five separate 2 L Erlenmeyer flasks. Each flask initially contained 800 mL of 100% ethanol, to which 200 mL of boiling water was added for a final 80% ethanol solution. Extraction was performed at 60°C for 30 min in a water bath, with manual agitation applied at 5‐min intervals. Following steeping, a Buchner funnel vacuum filtration system (Merck Millipore, Burlington, MA, USA), equipped with a 150 mm Whatman #4 filter paper, was used to filter the steeped tea extracts into a collection flask. Thereafter, the 80% ethanol filtrates were concentrated under vacuum with a Büchi Rotavapor V‐300 at 40°C (BÜCHI Labortechnik AG, Flawil, Switzerland).

#### 2.2.2 Solvent Partitioning and Amberlite XAD‐4 Column Chromatography

A 75 × 1180 mm glass column was packed with Amberlite XAD‐4 resin suspended in 25% ethanol‐water solution. Chromatographic separation was performed after dissolving 20 g of the unfermented rooibos extract in 50 mL of 50% ethanol: water (v/v) at 40°C followed by quantitative application to the column. The unfermented rooibos column was successively eluted with 25% ethanol: water (2 L), 50% ethanol: water (5 L), 75% ethanol: water (4.5 L), and finally 100% ethanol (2 L). A pre‐fraction of 1 L was collected, followed by several 150 mL fractions.

In contrast to the unfermented extract preparation, 20 g of the fermented rooibos extract was suspended in 50 mL of a 70% ethanol: water (v/v) solution at 40°C before quantitative application to the column. A stepwise gradient of decreasing polarity was used for elution at a flow rate of 10 mL/min. The elution was performed on the fermented rooibos column using an increasing ethanol gradient: 1.6 L of 25% ethanol: water, followed by 5 L of 50% ethanol: water, 4.5 L of 75% ethanol: water, and concluding with 2 L of 100% ethanol. A pre‐fraction of 600 mL was subsequently collected, followed by 150 mL fractions during the stepwise increase of the filtered ethanol concentrations as the eluting solvent. Column fractions were analyzed by reverse phase thin‐layer chromatography (RP‐TLC) to confirm the presence of aspalathin and nothofagin, with chloroform: methanol and sulfuric acid (6:3:1 v/v/v) as the development solvent. Visualization was achieved by spraying the plates with 0.5% *p*‐anisaldehyde in methanol: sulfuric acid: acetic acid (85:5:10) and heating at 120°C. Fractions containing compounds with similar Rf values (aspalathin and nothofagin) were combined and dried under vacuum with a Büchi Rotavapor V‐300 at 40°C.

#### 2.2.3 High‐Performance Liquid Chromatography

The main flavonoids in the organic unfermented and fermented rooibos extracts were quantified (de Beer et al. 2015) using high‐performance liquid chromatography (HPLC) at the Agricultural Research Council Infruitec‐Nietvoorbij (Stellenbosch, South Africa). The data were presented as g compound/100 g.

#### 2.2.4 Antioxidant Assays

The 2,2‐diphenyl‐1‐pircrylhydrazyl‐hydrate (DPPH) radical scavenging, 2,2′‐azino‐bis (3‐ethylbenzothiazoline‐6‐sulfonic acid) (ABTS) radical assay, ferric reducing antioxidant power (FRAP) assay, and oxygen radical absorbance capacity (ORAC) assay were conducted at the Oxidative Stress Research Centre (Cape Peninsula University of Technology, South Africa) based on the methods described by Rautenbach et al. (2010).

### 2.3 Cell Culture

Intestinal Porcine Epithelial Cells (IPEC‐J2) provided by BIOMIN (Vienna, Austria), intestinal columnar epithelial cells that were isolated from neonatal piglet mid‐jejunum (Berschneider 1989; Brosnahan and Brown 2012), were cultured in 1:1 (v/v) DMEM/F‐12 media. The culture media was enriched with 10% (v/v) FBS, 2.5 mM L‐glutamine, 1% (v/v) ITS, 16 mM HEPES, and 5 ng/mL EGF. Cells were incubated in a 95% humidified environment of 5% CO_2_ at 37°C LPS (10 μg/mL) was utilized to stimulate inflammation within the cells. Water‐soluble dexamethasone (a glucocorticoid) was optimized at a concentration of 0.15625 mM and employed as a positive control for anti‐inflammatory activity. All experiments were performed in triplicate (*n* = 3) and repeated to ensure consistency and reproducibility. The extract concentrations used in this study were based on ½ and ¼ IC_50_ results. For the experimental setup, IPEC‐J2 cells were seeded in white 96‐well plates (Costar) at a density of 1.5 × 10^4^ cells/well and incubated for 24 h to allow for attachment. The growth medium was then replaced with medium containing the various concentrations of the fermented and unfermented rooibos extracts and incubated for an additional 24 h. Following extract pretreatment, the extract‐containing medium was removed and replaced with medium supplemented with LPS (10 μg/mL). Cells were subsequently incubated with LPS for 24 h, after which cell viability, caspase activity, IL‐8 secretion, and transepithelial electrical resistance (TEER) were assessed. For gene expression analysis, cells were exposed to LPS (10 μg/mL) for 4 h only, after which total RNA was extracted for downstream analysis.

### 2.4 Cell Viability

Cell viability was assessed to confirm that all experimental conditions were maintained within non‐cytotoxic ranges. The CellTiter‐Glo Luminescent Cell Viability Assay (Promega, Southampton, UK) was used to determine the number of viable cells based on the amount of adenosine triphosphate (ATP) present and done according to the manufacturers' protocol. The ATP quantity was determined using the Veritas microplate luminometer (Turner Biosystems, Sunnyvale, California, USA).

### 2.5 Caspase Activity (Apoptosis)

Caspase 3/7 production was determined using the Caspase‐Glo 3/7 kit (Promega, Southampton, UK) according to the manufacturer's protocol. Caspase activity was measured using the Veritas microplate luminometer.

### 2.6 Real Time Quantitative Polymerase Chain Reaction Analysis

The IPEC‐J2 cells were grown in 35 mm petri dishes at 2.4 × 10^4^ cells/dish for 24 h, pre‐treated with various extract concentrations for 24 h and exposed to 10 μg/mL of LPS for 4 h. RNA was then extracted using the RNeasy mini kit (Qiagen, Hilden, Germany) according to the manufacturer's instructions. RNA concentration was determined using the Nanodrop 2000 spectrophotometer (Thermo Fisher Scientific, Wilmington, DE, USA). To produce complementary DNA (cDNA) 1 μg of total RNA was used for reverse transcription reaction using the iScript cDNA synthesis kit (Bio‐Rad Laboratories, Hercules, CA, USA) based on the manufacturer's protocol. The cDNA was used as a template for real‐time PCR amplification using the SsoAdvanced Universal SYBR Green Supermix (Bio‐Rad Laboratories, Hercules, CA, USA). Sample amplification was performed at 1 cycle at 95°C for 3 min, 40 cycles at 95°C for 10 s, 60°C for 30 s, and a melt cycle at 95°C for 15 s, 60°C for 1 min and 95°C for 15 s. The primers for all genes tested are listed in Table 1. Experiments were done in triplicate with three technical repeats, and data was quantified using a standard curve. The relative gene expression was calculated by using the comparative cycle threshold (2^−∆∆CT^) method (Livak and Schmittgen 2001) with porcine GAPDH as the housekeeping gene.

**TABLE 1:** Porcine primer sequences for real time quantitative polymerase chain reaction.

Gene	Primer sequence	References
Porcine *GAPDH* (Housekeeping gene)	Forward: TGGGCGTGAACCATGAGAA Reverse: CCTCCACGATGCCGAAGT	Gu et al. (2019)
Porcine *IL‐8*	Forward: GCTCTCTGTGAGGCTGCAGTT Reverse: AAGGTGTGGAATGCGTATTTATGC	
Porcine *IL‐6*	Forward: CAGGAACGAAAGAGAGCTCCAT Reverse: AAGGCAGTAGCCATCACCAGAA	
Porcine *TLR4*	Forward: ATCTGAGAGCTGGGACCCTT Reverse: ATGTGGGGATGTTGTCAGGG	Yan and Ajuwon (2017)
Porcine *Occludin*	Forward: CTACTCGTCCAACGGGAAAG Reverse: ACGCCTCCAAGTTACCACTG	
Porcine *ZO‐1*	Forward: AAGCCCTAAGTTCAATCACAATCT Reverse: ATCAAACTCAGGAGGCGGC	
Porcine *Claudin 4*	Forward: CTCTCGGACACCTTCCCAAG Reverse: GCAGTGGGAAGGTCAAAGG	

### 2.7 Inflammatory Cytokine Assay

Interleukin‐8 cytokine content in the supernatant of seeded cells was measured using the porcine ELISA kits (ELISA Duo set DY206, R&D Systems, Bio‐Techne, Minneapolis, MN, USA) based on the manufacturer's instructions. A standard curve was formed using porcine recombinant prepared in 1% bovine serum albumin (BSA) in DPBS (w/v) to the working concentration provided by the manufacturer (extracellular content was expressed as pg/mL). Sample absorbance was measured with the Synergy 2 Multi‐Mode Microplate Reader (BioTek, Winooski, Vermont, USA) at 450 nm. Data were analyzed by a standard (sigmoidal) curve produced from Gen5 Data Analysis Software (version 2 for Windows) and expressed as pg/mL.

### 2.8 Transepithelial Electrical Resistance (TEER) Analysis

The monolayer integrity of IPEC‐J2 cells was evaluated by measuring the transepithelial electrical resistance (TEER). The cells were seeded at 1 × 10^5^ cells on insert membranes (Trans well inserts with 12 mm diameter and 0.45 μm pore size) (Merck Millipore, Darmstadt, Germany). The monolayer integrity was monitored over 4 days using a Millicell ERS‐2 Voltohmmeter (Merck Millipore, Darmstadt, Germany) with the media replaced on each alternative day. When stable ohms' values were obtained, growth media was removed and cells were pre‐treated with the various tea extracts for 24 h. Cells were then exposed to 10 μg/mL LPS for 24 h and readings recorded the following day. TEER values were measured after every step, and the data were represented as Ohms/cm^2^.

### 2.9 ZO‐1 Immunostaining

Immunofluorescent staining was conducted as described by (Hui et al. 2020) with modifications. Cells were seeded in μ‐Slide 8‐well chamber slides (ibidi GmbH, Gräfelfing, Germany) at a cell density of 3 × 10^4^ cells/chamber for 24 h at 37°C. Samples were treated with the appropriate extract concentration and incubated for 24 h and then exposed to 10 μg/mL LPS for 24 h. The cells were fixed in 4% paraformaldehyde (PFA) for 10 min at RT and then washed with phosphate‐buffered saline supplemented with 0.1% Tween20 (PBST). The cells were blocked with a 10% goat serum blocking buffer for 1 h. Cells were then incubated with the ZO‐1 polyclonal antibody (#61‐7300, 1:100 dilutions, Thermo Fisher Scientific, Waltham, MA, USA) overnight at 4°C followed by three washes with PBST. The cells were then incubated for 1 h with a secondary Alexa Fluor 488 goat anti‐rabbit antibody (XF346494, 1:1000 dilution, Thermo Fisher Scientific, Waltham, MA, USA). Cells were subsequently washed three times with PBST and mounted using mounting medium containing 4′,6‐diamidino‐2‐phenylindole (DAPI) (#B2009, Santa Cruz Biotechnology, Dallas, TX, USA).

### 2.10 Statistical Analysis

All experiments had a minimum of three biological repeats and were repeated at least twice. Data was expressed as mean ± SEM. Statistical significance was confirmed using One‐way ANOVA followed by Tukey multiple comparison test. These tests were done using Graph Pad Prism 10 software. Significance was represented as various letters where *p*‐values between 0.05 and 0.0001 were accepted.

## 3 Results

### 3.1 Phenolic Characterization of Enriched Ethanolic Rooibos Extracts

To determine whether ethanolic extraction and XAD enrichment generated chemically distinct rooibos extracts, the total phenolic content and individual flavonoid composition of the unfermented and fermented extracts were quantified (Table 2). The enriched ethanolic extraction substantially increased the concentration of phenolic compounds in both rooibos types compared with the corresponding crude extracts, demonstrating the effectiveness of the extraction and enrichment procedure. Total phenolic content increased from 29.47 to 77.72 g compound/100 g soluble solids (SS) in the unfermented extract and from 20.17 to 53.10 g compound/100 g SS in the fermented extract. Despite enrichment of both extracts, the unfermented rooibos retained a markedly higher total phenolic content than the fermented rooibos, consistent with oxidative changes occurring during post‐harvest fermentation.

**TABLE 2:** Phenolic composition of the crude and enriched unfermented and fermented ethanolic rooibos extracts used in the study.

Compounds	Unfermented extract (g compound/100 g SS)		Percentage^a^ of enriched extract	Fermented extract (g compound/100 g SS)		Percentage^a^ of enriched extract
	Crude	Enriched	%	Crude	Enriched	%
S‐Eriodictyol‐6‐glc	nd	nd	0	1.496	4.57	8.61
R‐Eriodictyol‐6‐glc	nd	nd	0	1.498	4.56	8.59
S‐Eriodictyol‐8‐glc	nd	nd	0	0.312	0.908	1.71
R‐Eriodictyol‐8‐glc	nd	nd	0	0.359	1.02	1.92
PPAG	0.406	1.07	1.38	0.787	3.23	6.08
Aspalathin	20.187	60.0	77.20	1.480	9.46	17.82
Nothofagin	1.166	2.33	3	0.190	0.531	1
Isoorientin	2.388	4.95	6.37	4.308	10.6	19.96
Orientin	2.027	4.53	5.83	4.276	10.9	20.53
Bioquercetin	1.039	1.57	2.02	1.813	1.6	3.01
Vitexin	0.419	0.663	0.85	0.886	1.77	3.33
Hyperoside	0.342	0.419	0.54	0.605	0.873	1.64
Rutin	0.510	0.933	1.20	0.428	0.506	0.95
Isovitexin	0.450	0.637	0.82	0.905	1.51	2.84
Isoquercitrin	0.319	0.427	0.55	0.331	0.51	0.96
Luteoloside	0.213	0.213	0.27	0.499	0.575	1.08
Ferulic acid	nd	nd	0	nd	nd	0
Total	29.47	77.72		20.17	53.10	

Abbreviations: nd = not detected, SS = soluble solids, weight of extract after freeze‐drying.

^a^ Percentage in the extract for unfermented rooibos was calculated using the total of 77.72 g total polyphenols/100 g soluble solids and their percentage in extract for the fermented rooibos was calculated using the total 53.10 g total polyphenols/100 g soluble solids.

The phytochemical profiles of the enriched extracts also differed substantially. The enriched unfermented extract was characterized by a high abundance of aspalathin (60.0 g/100 g SS; 77.2% of the total phenolic composition), while eriodictyol glycosides were not detected. In contrast, the enriched fermented extract contained considerably lower aspalathin (9.46 g/100 g SS; 17.8%) but was enriched in orientin (20.5%), isoorientin (20.0%), and eriodictyol glycosides, reflecting the compositional changes associated with fermentation. These findings confirm that ethanolic extraction coupled with XAD enrichment generates concentrated polyphenol‐rich extracts while preserving the distinct phytochemical signatures of unfermented and fermented rooibos.

### 3.2 Antioxidant Capacity of Ethanolic Rooibos Extracts

To assess the antioxidant potential of the rooibos extracts, both unfermented and fermented samples were evaluated using radical scavenging assays namely 2,2‐azino‐bis (3‐ethylbenzothiazoline‐6‐sulfonic acid) (ABTS), 2,2‐diphenyl‐1‐picrylhydrazyl‐hydrate (DPPH), Ferric Reducing Antioxidant Power (FRAP) and Oxygen Radical Absorbance Capacity (ORAC) (Figure 1). In all assays performed, the unfermented rooibos extract exhibited significantly greater antioxidant activity compared to the fermented extract (*p* < 0.05). This enhanced activity is likely attributable to the higher levels of flavonoids and dihydrochalcones, particularly aspalathin, which are known to degrade during the fermentation process (Snijman et al. 2009). These findings are consistent with previous reports highlighting the superior radical‐scavenging capacity of unfermented rooibos due to its richer polyphenolic profile (Villaño et al. 2010). The strong antioxidant capacity of the unfermented extract may play a critical role in its protective effects against oxidative stress‐induced damage in intestinal epithelial cells.

**FIGURE 1:**
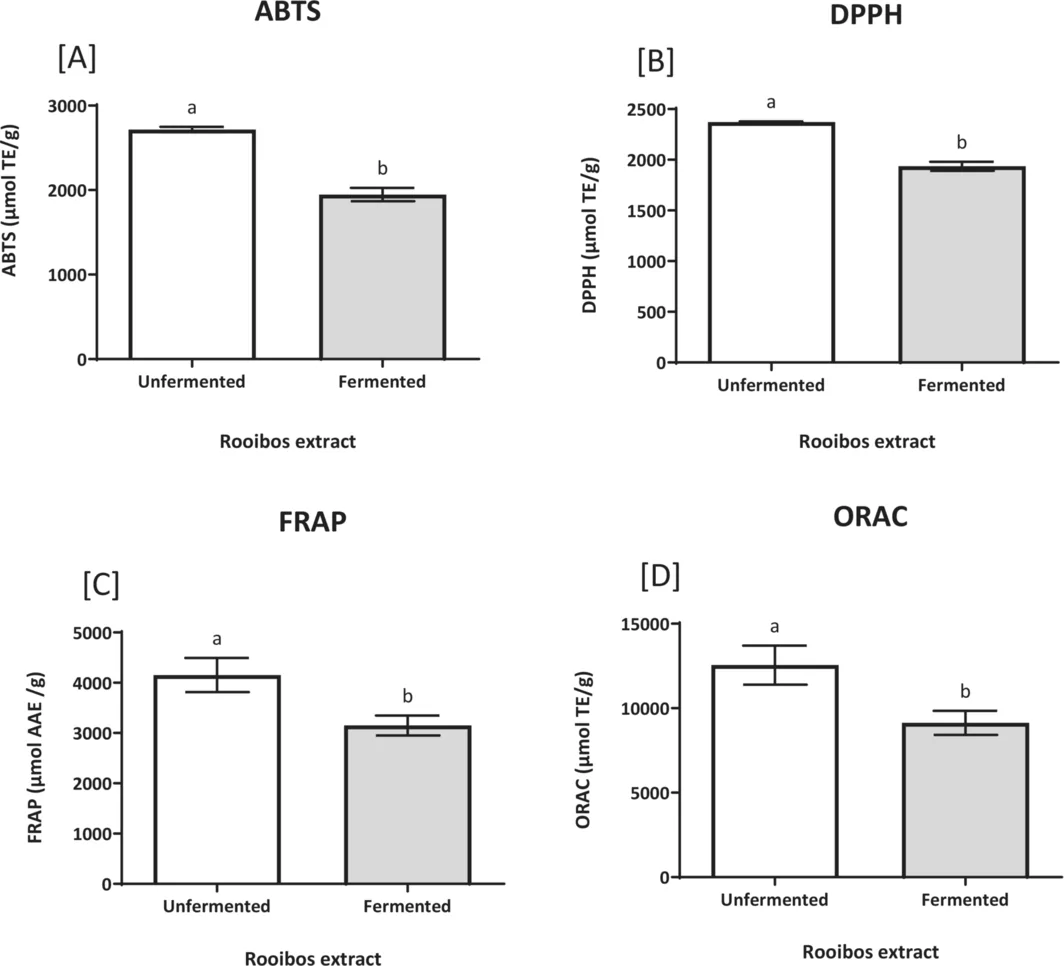
Antioxidant capabilities of the enriched unfermented and fermented rooibos extracts. (A) ABTS assay, (B) DPPH assay, (C) FRAP assay and (D) ORAC assay. Data was presented as mean ± SEM, *n* = 3. Statistical significance was indicated by different letters whilst common letters indicated no significance was present between samples. Significance was seen at *p* < 0.05. ABTS = (2,2′‐azino‐bis (3‐ethylbenzothiazoline‐6‐sulfonic acid)), DPPH = (2,2‐diphenyl‐1‐picryl‐hydrazyl‐hydrate), FRAP = Ferric Reducing Antioxidant Power, ORAC = Oxygen Radical Absorbance Capacity.

### 3.3 IC50 Determination of the Ethanolic Rooibos Extracts

To determine the half maximal inhibitory concentration (IC_50_) of each extract, an ATP‐based cell viability assay was performed to assess the dose‐dependent response of the cells to the treatments. Extract concentrations for downstream analysis were selected based on the IC_50_ for both the UF and F extracts. Although all concentrations of the unfermented extract resulted in significantly reduced ATP production compared to the control (Figure 2A), only the 3.2 mg/mL concentration decreased ATP levels to below 70%. This suggests that IPEC‐J2 cells tolerated concentrations up to 1.6 mg/mL, maintaining more than 70% cell viability. In contrast, IPEC‐J2 cells exhibited markedly lower tolerance to the fermented extract, as concentrations as low as 0.4 mg/mL induced pronounced cytotoxicity, reducing cell viability to below 50%. Concentrations of 0.2 mg/mL and lower maintained ATP production above 70%, indicating acceptable cell viability at these levels. Based on the IC_50_ values and to ensure the use of non‐cytotoxic concentrations, subsequent experiments were conducted using 1.0, 0.5, and 0.25 mg/mL for the unfermented extract, and 0.2, 0.1, and 0.05 mg/mL for the fermented extract.

**FIGURE 2:**
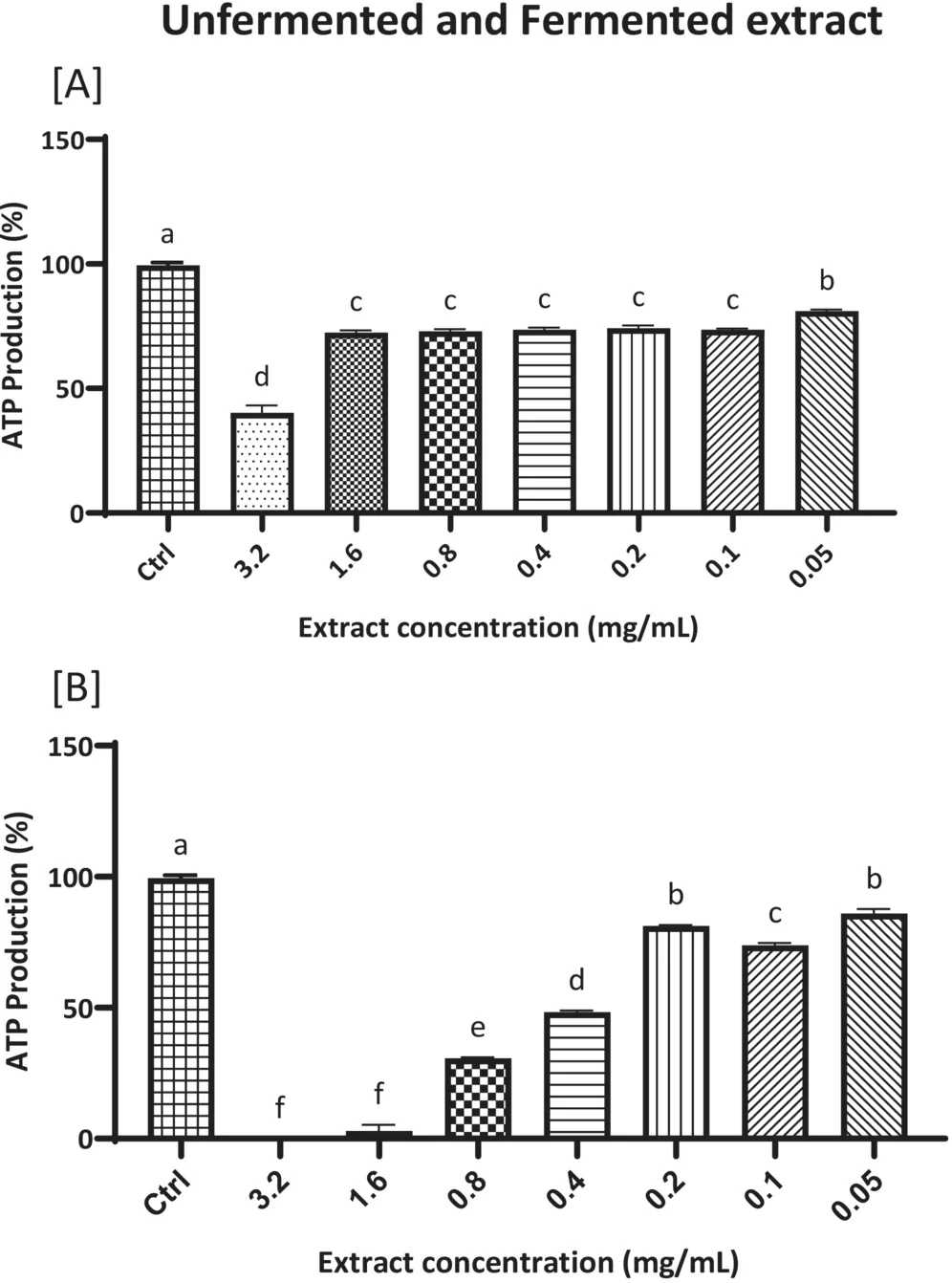
ATP dose response of IPEC‐J2 cells to the pre‐treatment with (A) unfermented and (B) fermented ethanolic rooibos extracts. Data was presented as mean ± SEM, *n* = 3. Statistical significance was indicated by different letters while common letters indicated no significance was present between samples. Significance was seen at *p* < 0.05.

### 3.4 The Protective Effect of Ethanolic Rooibos Extracts on Cell Viability and Apoptosis in LPS‐Induced Inflammation

Cell viability reflects the normal physiological function of cells, including essential metabolic processes such as ATP production and the regulation of apoptosis. To evaluate the protective potential of the ethanolic rooibos extracts, the effects of unfermented and fermented extracts on cell viability and apoptosis were assessed in IPEC‐J2 cells exposed to LPS‐induced inflammation. Figure 3A shows that 10 μg/mL LPS significantly (*p* = 0.0157) decreased the ATP production to 86.72% compared to the control. Dexamethasone (0.15625 mM), unfermented extract at 0.5 mg/mL, and fermented extract at 0.1 mg/mL did not significantly affect ATP production relative to the LPS‐only treatment. Considering that sub‐toxic levels are defined as maintaining ≥ 70% viability, both the unfermented extract at 0.25 mg/mL (*p* = 0.0055) and the fermented extract at 0.2 mg/mL (*p* = 0.0054) resulted in a significant reduction in ATP production yet remained above the 70% threshold. In contrast, the highest concentration of the unfermented extract (1.0 mg/mL) and the lowest concentration of the fermented extract (0.05 mg/mL) caused a significant decline in ATP production compared to the control, with values falling below the 70% viability limit. Figure 3B illustrates the effects of extracts on the caspase 3/7 activity. Assay validity was confirmed using IPEC‐J2 cells exposed to 5% DMSO, which exhibited a significant increase in caspase‐3/7 activity (*p* < 0.0001), indicating effective induction of apoptosis under cytotoxic conditions. All three concentrations (0.2, 0.1, and 0.05 mg/mL) of the fermented extract significantly (*p* < 0.0001) elevated caspase activity compared to the LPS‐only exposure. In contrast, cells exposed to LPS alone showed no significant difference from the control group, indicating that LPS exposure did not markedly alter caspase activity under these conditions. Similarly, the unfermented extract, across all tested concentrations, exhibited no significant effect on caspase activity relative to the LPS‐treated cells.

**FIGURE 3:**
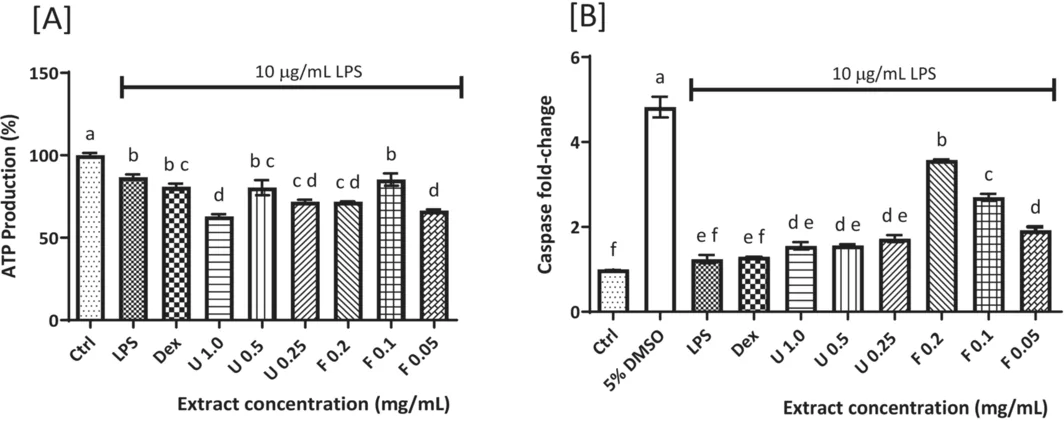
The effects of unfermented (U) and fermented (F) ethanolic rooibos extracts on the (A) ATP and (B) Caspase 3/7 production. Data was presented as mean ± SEM, *n* = 3. Statistical significance was indicated by different letters whilst common letters indicated no significance was present between samples. Significance was seen at *p* < 0.05.

### 3.5 The Protective Effect of Ethanolic Rooibos Extracts on IL‐8 Protein Expression in LPS‐Induced Inflammation

To evaluate the anti‐inflammatory potential of the ethanolic rooibos extracts, the levels of the pro‐inflammatory cytokine interleukin‐8 (IL‐8) were quantified in IPEC‐J2 cells (Figure 4). Initially, it was observed that cells treated with 10 μg/mL LPS exhibited a significant increase (*p* < 0.001) in IL‐8 protein expression compared to the control, confirming the successful induction of an inflammatory response. Cells pre‐treated with the unfermented extract at 0.25 mg/mL and the fermented extracts at 0.1 and 0.05 mg/mL exhibited no significant reduction in IL‐8 concentrations. In contrast, treatment with dexamethasone (*p* < 0.0001), unfermented extract at 1.0 mg/mL (U 1.0; *p* < 0.0001), unfermented extract at 0.5 mg/mL (U 0.5; *p* < 0.05), and fermented extract at 0.2 mg/mL (F 0.2; *p* < 0.05) resulted in a significant downregulation of IL‐8 levels compared to the LPS‐stimulated control. Interestingly, the extracts demonstrated a dose‐dependent pro‐inflammatory trend, as lower concentrations were associated with elevated IL‐8 secretion.

**FIGURE 4:**
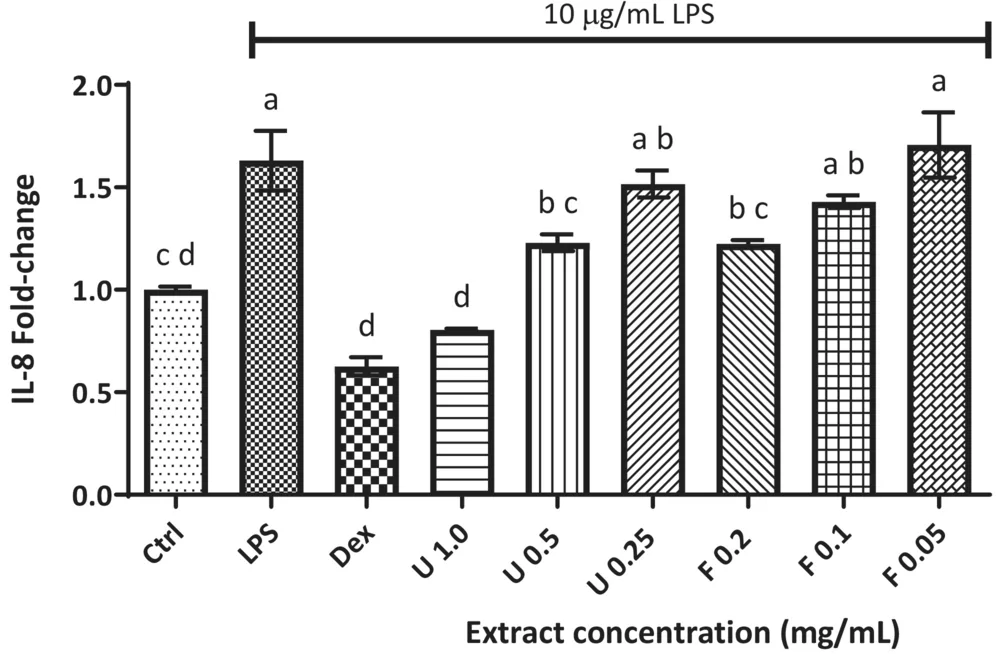
The effect of pre‐treatment with unfermented (U) and fermented (F) rooibos extracts on IL‐8 protein production (pg/mL) in LPS‐stimulated IPEC‐J2 cells. Data was presented as mean ± SEM, *n* = 3. Statistical significance was indicated by different letters whilst common letters indicated no significance was present between samples. Significance was seen at *p* < 0.05.

### 3.6 The Protective Effect of Ethanolic Rooibos Extracts on Cytokine Gene Expression

To further substantiate the anti‐inflammatory potential of the ethanolic extracts, the gene expression levels of key pro‐inflammatory mediators, including *IL‐8*, *IL‐6*, and the *TLR4* receptor, were evaluated. In Figure 5A–C, LPS significantly (*p* < 0.0001) upregulated the gene expression of each pro‐inflammatory cytokine showing the strong elicited inflammatory response. Figure 5A illustrates that all concentrations of the unfermented (U) extract significantly downregulated *IL‐8* gene expression, with U 0.5 mg/mL (*p* < 0.0001) exhibiting the most pronounced anti‐inflammatory effect. Additionally, both the highest and lowest concentrations of the fermented (F) extract significantly downregulated *IL‐8* expression (*p* < 0.0001), whereas pre‐treatment with F 0.1 mg/mL showed no significant effect on *IL‐8* expression in LPS‐induced IPEC‐J2 cells. Figure 5B shows that both dexamethasone and F 0.1 mg/mL had no significant effect on *IL‐6* gene expression in LPS‐induced cells, whereas pre‐treatment with F 0.2 mg/mL significantly increased *IL‐6* expression following LPS exposure. In contrast, similar to the trend observed for *IL‐8*, all concentrations of the unfermented (U) extract, as well as fermented extract at 0.05 mg/mL, significantly (*p* < 0.0001) downregulated *IL‐6* gene expression. The *TLR4* gene expression was significantly (*p* < 0.0001) downregulated by all concentrations of both extracts (Figure 5C).

**FIGURE 5:**
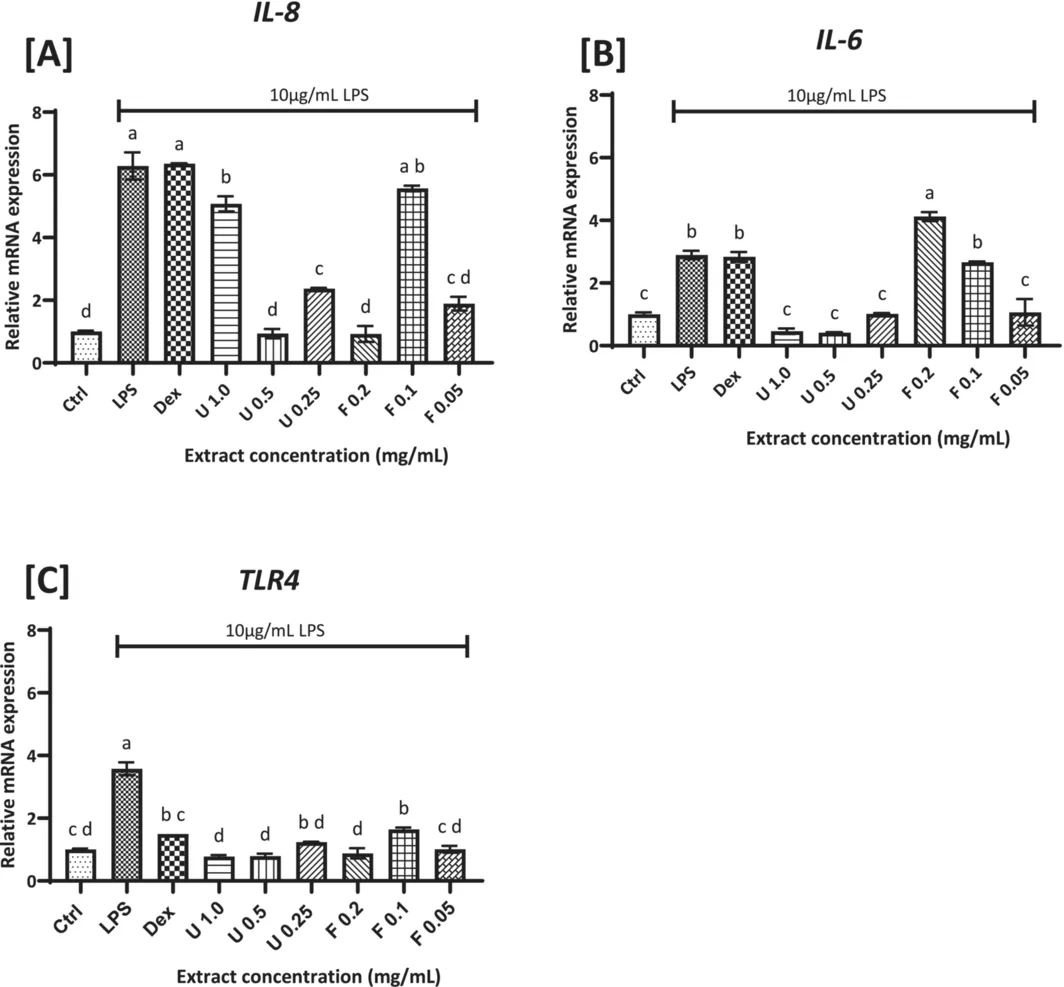
Gene expression analysis of pro‐inflammatory markers (A) *IL‐8*, (B) *IL‐6*, and (C) *TLR4* in LPS‐stimulated IPEC‐J2 cells pre‐treated with unfermented (U) and fermented (F) rooibos extracts for 24 h followed by 4 h exposure to 10 μg/mL LPS. Data were presented as mean ± SEM, *n* = 3. Statistical significance was indicated by different letters, whilst common letters indicated no significance was present between samples. Significance was seen at *p* < 0.05.

### 3.7 The Barrier Protective Effect of Ethanolic Rooibos Extracts on Tight Junction Gene Expression

At the intestinal barrier, tight junctions play a crucial role in maintaining epithelial integrity and selective permeability. As inflammation is known to disrupt tight junction structure and function, the expression of *Claudin‐4*, *Occludin*, and *ZO‐1* was evaluated to determine whether ethanolic rooibos extracts modulate tight junction–related gene expression (Figure 6). The results demonstrated that LPS exposure led to a slight reduction in *ZO‐1* gene expression (Figure 6A). Conversely, treatment with all concentrations of the unfermented rooibos extracts (U 1.0; *p* < 0.0001, U 0.5; *p* < 0.001, U 0.25; *p* < 0.001) significantly upregulated *ZO‐1* expression, surpassing that of the untreated control. When evaluating the fermented rooibos extract, the F 0.2 mg/mL concentration showed no significant protective effect on the cells. However, treatments with F 0.1 mg/mL and F 0.05 mg/mL significantly upregulated *ZO‐1* gene expression, suggesting potential barrier‐protective effects. LPS did not have a significant effect on the downregulation of *Occludin* (Figure 6B), a slight increase was observed, although not significant. All concentrations of the unfermented rooibos extract (U) (*p* < 0.05), as well as the fermented extract at 0.2 mg/mL (*p* ≤ 0.0025), significantly increased *Occludin* gene expression compared to the control cells. The other concentrations of the fermented extract (0.1 and 0.05 mg/mL) induced an upregulation of *Occludin* expression, although the changes were not statistically significant. A significant upregulation of *Claudin‐4* gene expression was observed only at F 0.1 mg/mL (*p* < 0.0001). Although increases were noted at concentrations U 0.5 mg/mL, F 0.2 mg/mL, and F 0.05 mg/mL, these changes were not statistically significant.

**FIGURE 6:**
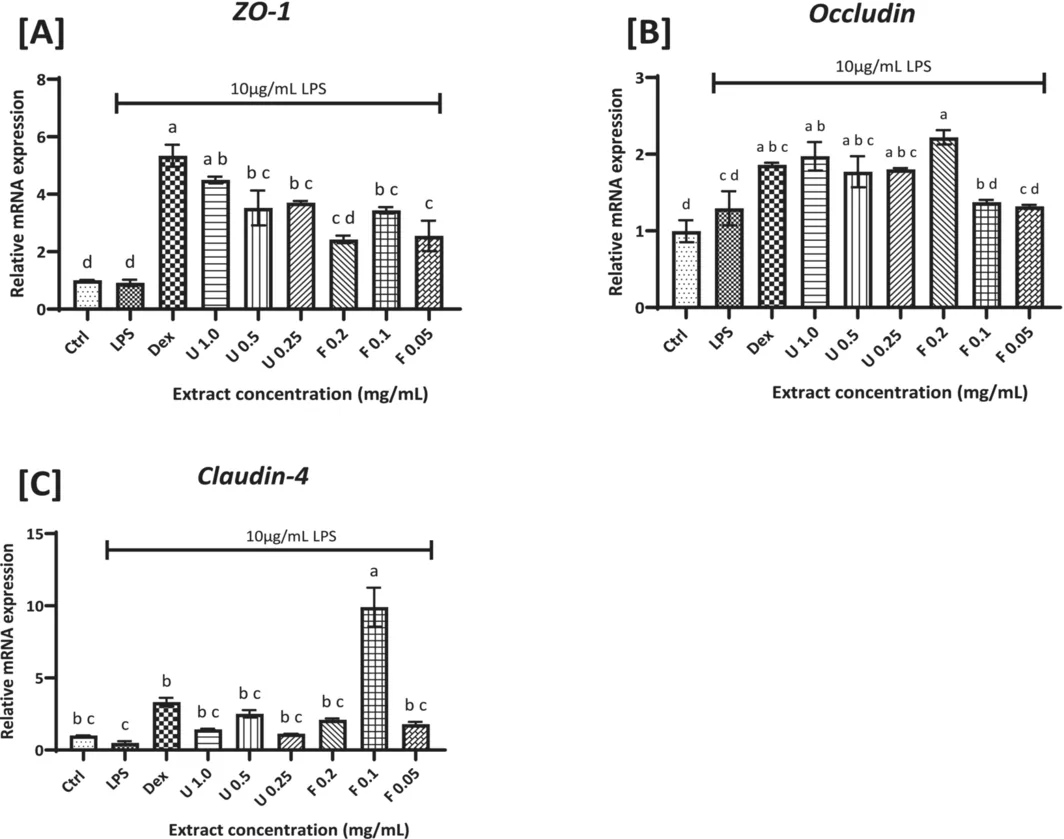
Gene expression of tight junction proteins (A–C) *ZO‐1*, (B) *Occludin*, and (C) *Claudin‐4* in IPEC‐J2 cells pre‐treated with ethanolic unfermented or fermented rooibos extracts for 24 h, followed by exposure to 10 μg/mL LPS for 4 h. Data were presented as mean ± SEM, *n* = 3. Statistical significance was indicated by different letters while common letters indicated no significance was present between samples. Significance was seen at *p* < 0.05.

### 3.8 Effects of Ethanolic Rooibos Extracts on Transepithelial Electrical Resistance (TEER)

The integrity of the intestinal barrier is indicative of proper tight junction function, which is essential for regulating paracellular permeability. Transepithelial electrical resistance (TEER) measurements were performed to evaluate whether ethanolic rooibos extracts exert a protective effect on the IPEC‐J2 epithelial barrier under LPS‐induced inflammatory conditions. Results presented in Figure 7 indicate that IPEC‐J2 cells exposed to 10 μg/mL LPS (*p* ≤ 0.0347) exhibited a significant reduction in the barrier integrity compared to the control. Pre‐treatment with U 0.5 mg/mL, F 0.1 mg/mL, and F 0.05 mg/mL resulted in a significant increase in TEER values compared to LPS‐exposed cells, indicating protection of the epithelial barrier. The TEER values in these treatment groups were comparable to those of the control cells, suggesting restoration of barrier integrity. In contrast, the remaining concentrations, U 1.0 mg/mL (*p* < 0.05), U 0.25 mg/mL (*p* ≤ 0.0017), and F 0.2 mg/mL (*p* < 0.0001), showed a significant decrease in TEER values compared to the control cells. These values were comparable to those of the LPS‐exposed group, suggesting a lack of protective effect and consequently reduced barrier integrity. The F 0.2 mg/mL treatment exhibited the least protective effect, showing a significant decrease in TEER values. Notably, U 0.5, F 0.1, and F 0.05 mg/mL demonstrated greater significant efficacy than dexamethasone in preserving barrier integrity under LPS‐induced inflammatory conditions.

**FIGURE 7:**
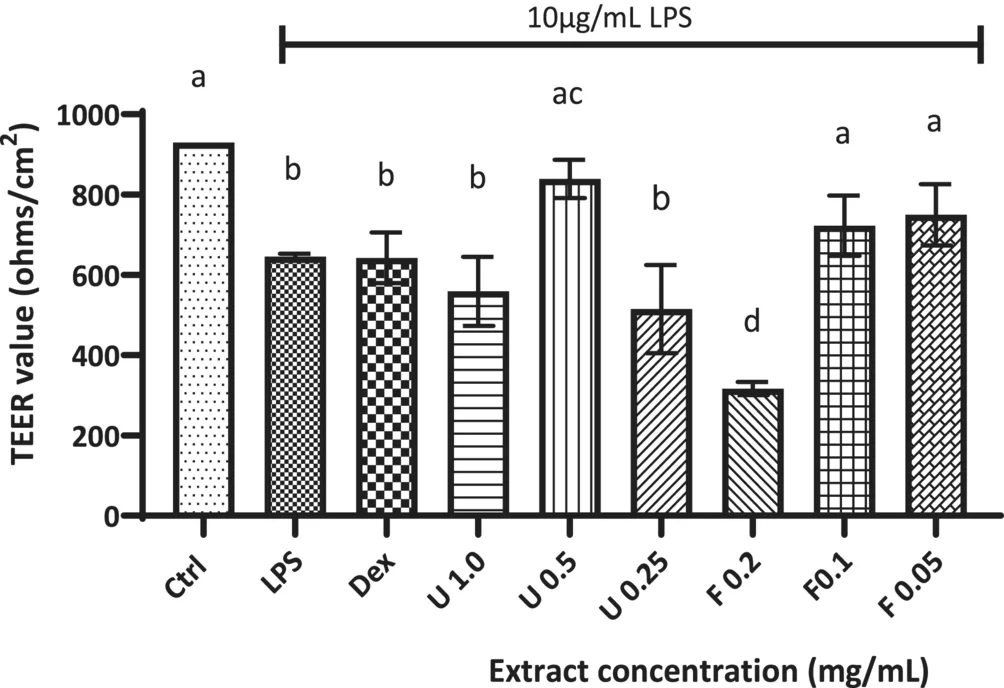
Effects of ethanolic unfermented and fermented rooibos extracts on transepithelial electrical resistance (TEER) in IPEC‐J2 cells stimulated with 10 μg/mL LPS for 24 h. Cells were pre‐treated with the respective extracts for 24 h prior to LPS exposure. Data was presented as mean ± SEM, *n* = 3. Statistical significance was indicated by different letters whilst common letters indicated no significance was present between samples. Significance was seen at *p* < 0.05.

### 3.9 Effect of Ethanolic Rooibos Extracts on ZO‐1 Localization in IPEC‐J2 Cells

Immunofluorescence analysis was performed to evaluate the effects of the ethanolic unfermented and fermented rooibos extracts on the expression and localisation of the tight junction protein ZO‐1 in LPS‐stimulated IPEC‐J2 cells (Figure 8). Representative fluorescence images demonstrated strong, continuous ZO‐1 staining along the intercellular junctions of the untreated control cells, whereas exposure to 10 μg/mL LPS markedly reduced ZO‐1 fluorescence intensity and disrupted junctional localisation, indicating impaired epithelial barrier integrity. Pre‐treatment with dexamethasone restored ZO‐1 expression to levels comparable with the untreated control. Similarly, both the unfermented and fermented ethanolic rooibos extracts increased ZO‐1 immunofluorescence compared with the LPS‐treated cells, although the magnitude of the response differed between the extracts and concentrations tested. The unfermented extract produced the greatest increase in ZO‐1 expression at 0.5 mg/mL, with the highest relative fluorescence intensity (114.5 RFU), followed by 0.25 mg/mL (87.3 RFU) and 1.0 mg/mL (77.6 RFU). In contrast, the fermented extract produced a more moderate restoration of ZO‐1 expression, with relative fluorescence values ranging from 53.7 to 66.4 RFU across the concentrations evaluated. Overall, the unfermented extract exhibited a greater capacity to restore ZO‐1 protein expression than the fermented extract, with the most pronounced effect observed at 0.5 mg/mL. These findings are consistent with the improved epithelial barrier protection observed following treatment with the ethanolic rooibos extracts and suggest that the enhanced barrier‐protective activity of the unfermented extract may be associated with its higher polyphenol and aspalathin content.

**FIGURE 8:**
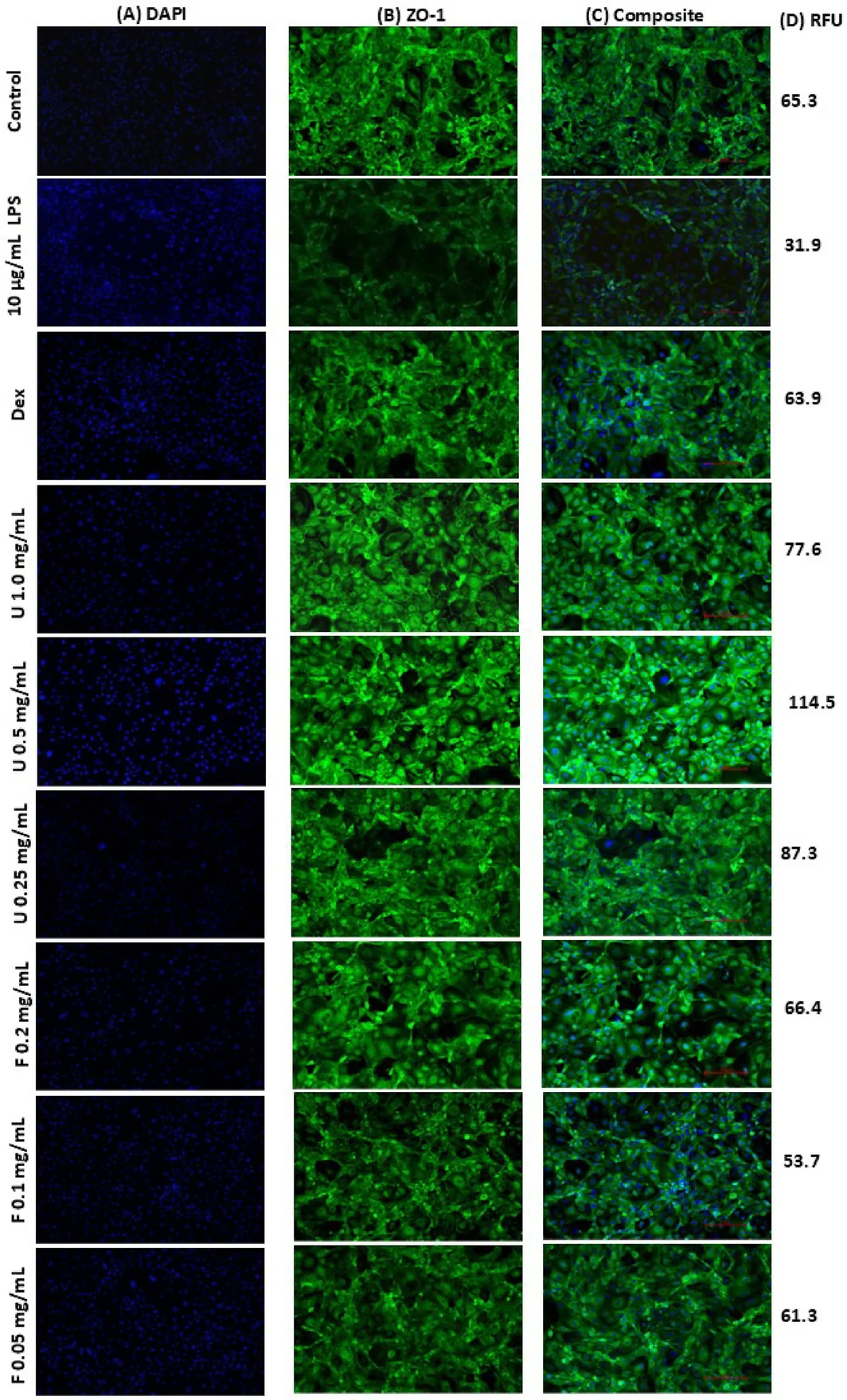
Effects of ethanolic unfermented and fermented rooibos extracts on ZO‐1 protein expression in IPEC‐J2 cells stimulated with 10 μg/mL LPS for 24 h. Cells were pre‐treated with the respective extracts for 24 h prior to LPS exposure. The immunofluorescence images of IPEC‐J2 cells show (A) DAPI‐stained nuclei, (B) ZO‐1 localization at the cell junctions, (C) the merged composite of both 495 channels and (D) the relative fluorescent units (RFU) of the ZO‐1 staining as determined using ImageJ 496 software. Representative fluorescence images illustrate ZO‐1 localization and abundance.

## 4 Discussion

Rooibos is an indigenous South African plant that has been studied extensively for its diverse health‐promoting properties (Afrifa et al. 2023), primarily attributed to its unique polyphenolic profile (Ku et al. 2015; Magcwebeba et al. 2016). The total polyphenolic content and flavonoid composition of rooibos extracts depend on multiple factors including harvesting technique (Turkmen et al. 2009), extraction solvent (Mohammed et al. 2022), temperature (Santos et al. 2016), and extraction method (Shaukat et al. 2023). Extraction is therefore a critical step in phytochemical content, as solvent polarity directly influences solubility, yield, and ultimately bioactivity of polyphenols (Gil‐Martín et al. 2022).

Fermentation is an important traditional processing step in rooibos production and is primarily undertaken to develop the characteristic red‐brown color, aroma, and flavor associated with conventional rooibos tea. During this process, enzymatic and non‐enzymatic oxidation alters the native phenolic profile of the plant, particularly the dihydrochalcones aspalathin and nothofagin, resulting in the formation of flavanone C‐glucosides and other oxidation products (De Beer et al. 2019). The higher antioxidant capacity reported for unfermented rooibos compared with traditionally processed rooibos (Von Gadow et al. 1997; Standley et al. 2001) stimulated the development of green rooibos as an alternative rooibos product (De Beer and Joubert 2002). Green rooibos is processed to minimize oxidative changes and thereby preserve its green color and higher levels of aspalathin and other phenolic compounds. This is achieved through rapid drying or enzyme‐inactivation approaches, including steaming, which limit oxidation of the freshly harvested plant material. Consequently, fermented and unfermented rooibos should not be considered chemically equivalent products, as fermentation produces distinct phytochemical profiles with potentially different biological activities. In the present study, unfermented rooibos retained substantially higher levels of total phenolics and aspalathin and demonstrated greater antioxidant capacity than fermented rooibos, supporting previous observations that the antioxidant potential of rooibos is reduced following fermentation (Joubert et al. 2004). However, fermentation should not necessarily be regarded as detrimental to all aspects of rooibos bioactivity. The formation of structurally distinct flavonoid metabolites may confer biological properties that differ from those of the parent compounds, such as the Eriodictyol‐6‐glc and ‐8‐glc products, and in the present study the fermented extract demonstrated effective barrier‐protective and anti‐inflammatory activity at lower concentrations for selected endpoints. Thus, while unfermented rooibos may offer advantages where preservation of aspalathin and antioxidant capacity is desired, fermented rooibos remains a valuable product because fermentation generates a distinct phytochemical composition and may provide complementary biological activities.

The chemical structure of polyphenols, defined by functional groups such as hydroxyl, methoxyl, and glycosidic moieties, determines both polarity and solubility, thereby influencing biological activity (Haminiuk et al. 2014; Palaiogiannis et al. 2023). Polar compounds dissolve readily in aqueous solvents, whereas less polar phenolics require moderately polar or non‐polar solvents for efficient extraction (Tripathi et al. 2025). Ethanol is widely used as an effective, food‐safe solvent due to its intermediate polarity and ability to form hydrogen bonds with hydroxyl, carboxyl, and aromatic groups in polyphenols (Cano‐Avendaño et al. 2021; Henkel et al. 2018; Musa et al. 2011). Ethanol‐water mixtures often enhance extraction efficiency and antioxidant activity compared to other solvent systems (Lapornik et al. 2005; Yeasmen and Islam 2015). Plaskova and Mlcek (2023) similarly reported that both water and ethanol‐water mixtures are effective and environmentally sustainable, with water extracting highly polar compounds and ethanol improving recovery of less polar flavonoids, thus broadening extract diversity.

In the present study, ethanol extraction and column enrichment were not employed simply as an alternative solvent, but as a strategy to generate a chemically distinct rooibos extract with an altered flavonoid composition for biological evaluation. Unlike conventional aqueous extraction, 80% ethanol combined with Amberlite XAD‐4 fractionation preferentially recovered and concentrated flavonoids, particularly the bioactive dihydrochalcones aspalathin and nothofagin, producing extracts with substantially higher polyphenol concentrations than those previously obtained using aqueous extraction (De Bruyn‐Orr et al. 2025). Consequently, the present study was designed to determine whether modification of the phytochemical profile through extraction methodology translates into altered biological activity, thereby addressing an important question in the development of standardized rooibos nutraceuticals. Consistent with previous reports, fermentation markedly altered the phytochemical composition of rooibos through oxidative conversion of aspalathin into flavone derivatives (Erlwanger and Ibrahim 2017). The enriched unfermented ethanolic extract contained 77.72 g compounds/100 g solids, compared with 53.10 g compounds/100 g solids in the fermented extract, with aspalathin comprising 77.20% of the unfermented extract but only 17.82% of the fermented extract. In contrast, orientin (20.53%) and isoorientin (19.96%) became the predominant flavonoids in the fermented extract, confirming that post‐harvest processing substantially remodels the flavonoid profile (Joubert, Richards, et al. 2008; De Bruyn‐Orr et al. 2025). Importantly, comparison with our previous aqueous extraction study demonstrates that ethanol extraction produced a markedly greater recovery of polyphenols after XAD enrichment. The enriched unfermented ethanolic extract contained almost four times more total polyphenols than the corresponding aqueous extract (77.72 vs. 20.73 g/100 g solids), while the enriched fermented ethanolic extract contained nearly ten‐fold higher concentrations than the aqueous fermented extract (53.10 vs. 5.38 g/100 g solids). These differences demonstrate that solvent selection profoundly influences the chemical composition of rooibos extracts and therefore the phytochemical dose delivered during biological testing.

The antioxidant assays (ABTS, DPPH, FRAP, and ORAC) demonstrated that unfermented enriched ethanol rooibos extract possesses significantly higher antioxidant activity compared to the fermented extract. The reduction in antioxidant capacity following fermentation is consistent with previous reports suggesting that oxidative and enzymatic processes during fermentation tea processing led to the degradation or structural modification of polyphenolic compounds, particularly aspalathin and other flavonoids, which contribute significantly to the antioxidant potential of rooibos. The higher radical scavenging and reducing power observed in the unfermented extract therefore reflects its richer polyphenolic profile and stronger capacity to neutralize reactive species. This trend was also evident in the aqueous extracts, where the unfermented sample exhibited stronger antioxidant activity than the fermented extract, consistent with its higher polyphenolic content (De Bruyn‐Orr et al. 2025). These findings highlight the importance of processing conditions in preserving the bioactive properties of rooibos and suggest that unfermented rooibos may offer greater protective effects in biological systems where oxidative stress is implicated.

With the rapid expansion of phytomedicine and the growing development of nutraceuticals and dietary supplements, it has become increasingly important to evaluate both the efficacy and safety of these bioactive compounds. While polyphenols and flavonoids are widely recognized for their beneficial effects on human health, a comprehensive understanding of their mechanisms of action, bioavailability, and potential adverse effects remains essential to ensure their safe and effective use (Wachira et al. 2024). Optimizing phytochemical concentration is particularly important for achieving therapeutic efficacy while avoiding cytotoxicity. The dose response profile shown in Figure 2 illustrates the variation in IPEC‐J2 cell tolerance to both unfermented and fermented enriched rooibos extracts. The dose–response profiles observed in this study demonstrate that the chemically enriched ethanolic rooibos extracts exhibited distinct concentration‐dependent effects in IPEC‐J2 cells, highlighting that increased phytochemical recovery does not necessarily translate into proportionally greater biological benefit. The unfermented extract was well tolerated over a broader concentration range, whereas the fermented extract displayed a narrower therapeutic window despite being effective at lower concentrations. These differences are likely attributable to fermentation‐induced alterations in flavonoid composition, together with the higher polyphenol concentrations achieved through ethanolic extraction and XAD enrichment (Joubert, Richards, et al. 2008; Standley et al. 2001). A lower aspalathin content in the fermented extract may decrease its antioxidant capability (Vhangani et al. 2022), thereby contributing to reduced cellular tolerance at equivalent concentrations. It could therefore be predicted that increasing the extraction or preservation of key flavonoids such as aspalathin may enhance the overall biological benefits of the extract (Mohammed et al. 2022).

Lipopolysaccharide (LPS), a major component of the outer membrane of Gram‐negative bacteria, is well known for its deleterious effects on various cell types. At elevated concentrations, LPS can trigger pronounced inflammatory responses, leading to increased rates of apoptosis and, in severe cases, cell death (Kell and Pretorius 2015). These adverse effects then lead to disruptions of the intestinal barrier, increasing the permeability and potentially leading to inflammation (Nighot et al. 2017). Consequently, LPS serves as a well‐established model compound for inducing and studying inflammation in vitro. Phytochemicals are well known to modulate a variety of cellular processes (Upadhyay and Dixit 2015), and their biological effects are closely associated with their concentration and bioavailability (Kuwahara et al. 2017; Spissu et al. 2023). In this study, the protective effects of ethanolic extracts of unfermented and fermented rooibos were investigated, resulting in an enriched extract with potentially enhanced bioactive properties. Exposure to the unfermented extract at 1 mg/mL resulted in a significant reduction in ATP production. This observation may be attributed to the relatively high extract concentration, which, in combination with LPS‐induced cellular stress, could disrupt mitochondrial function and other essential cellular processes. Polyphenolic phytochemicals have previously been reported to inhibit the F₀F₁‐ATPase complex, a critical regulator of mitochondrial ATP synthesis (Zheng and Ramirez 2000). A comparable trend was observed following treatment with the fermented extract at 0.05 mg/mL, which also led to a significant decrease in ATP production. This reduction may reflect an insufficient extract concentration to confer a protective biological effect under LPS‐induced inflammatory conditions (Muller et al. 2018). As anticipated, treatment with LPS at 10 μg/mL alone resulted in decreased ATP production. Nevertheless, both the unfermented (U 0.5; U 0.25) and fermented extracts (F 0.2; F 0.1) maintained ATP levels or preserved cell viability above 70%, indicating minimal cytotoxicity under the tested conditions.

The evaluation of caspase‐3/7 activity revealed that pre‐treatment with the unfermented ethanolic rooibos extract did not result in a significant increase in apoptotic activity compared to the LPS‐treated control. However, apoptosis was significantly elevated in the cells treated with the fermented ethanolic rooibos extract. Several factors may account for the higher fold‐increase in activity observed in cells pre‐treated with fermented tea, one of which is the alteration of bioactive compounds resulting from the fermentation process. During the fermentation process (oxidation), some of these primary polyphenols, particularly aspalathin and nothofagin, are oxidized and transformed into other compounds, such as R‐Eriodictyol‐6‐glc and other sugars that are not present in unfermented rooibos (Muller et al. 2018). These novel compounds and changes in the ratio of existing compounds may potentially exert a pro‐apoptotic effect in certain cell types. Additionally, the fermentation process leads to an overall decrease in antioxidant activity compared to unfermented rooibos. Therefore, lower polyphenol content as well as the appearance or formation of oxidized polyphenols can affect antioxidant capacity, which might reach a threshold or interact differently with cellular pathways in intestinal cells to promote a pro‐oxidant effect and subsequent caspase activation (Rudrapal et al. 2022). A study by Zhao et al. (2011) which investigated a different tea type, Pu‐erh, a post‐fermented tea, reported that the fermentation process can enhance its anti‐cancer properties and increase caspase activity in cancer cells.

At the intestinal barrier, an inflammatory response against irritants such as LPS will be elicited by the epithelial cells. This response is mediated through the activation of lipopolysaccharide (LPS)‐responsive inflammatory receptors particularly Toll‐like receptor 4 (TLR4), resulting in the production and release of pro‐inflammatory cytokines, including interleukin‐6 (IL‐6), interleukin‐8 (IL‐8), and tumor necrosis factor‐α (TNF‐α) (Semin et al. 2021; Zhang et al. 2025). The LPS‐TLR4 interaction induces a cascade of complex intracellular signaling including Mitogen‐Activated Protein Kinase (MAPK), TIR‐domain–containing adaptor‐inducing interferon‐β (TRIF), and Myeloid Differentiation Primary Response 88 (MyD88) signaling pathways, which lead to the release of pro‐inflammatory cytokines such as IL‐8 and IL‐6 (Page et al. 2022). IL‐8 is a well‐known chemokine that plays an initial role in recruiting immune cells to the inflamed site (Russo et al. 2014). IL‐6 is a cytokine with multiple functional cytokines involved in regulating both chronic and acute inflammation due to LPS‐induced inflammation of intestinal cells (Matsuda 2023). In this study treatment with LPS significantly upregulated the gene expression of *IL‐8*, *IL‐6*, and *TLR4* as well as IL‐8 protein production compared to the untreated control, confirming successful induction of an inflammatory response in IPEC‐J2 cells. Dexamethasone, used as a positive anti‐inflammatory control, significantly reduced the gene expression of *TLR4* and IL‐8 protein production, consistent with its known inhibitory effects on pro‐inflammatory signaling. Both unfermented and fermented rooibos extracts demonstrated concentration‐dependent anti‐inflammatory effects, although their efficacy varied between cytokines. The unfermented extract notably reduced *IL‐8* and *TLR4* gene expression at higher concentrations (0.5 mg/mL), suggesting significant suppression of chemokine and receptor‐mediated inflammatory pathways. The fermented extract was effective at the lower concentration (0.05 mg/mL) in downregulating *IL‐6* and *IL‐8*, indicating enhanced bioactivity possibly due to fermentation‐associated chemical modifications. Both the unfermented and fermented rooibos extracts significantly reduced *TLR4* gene expression at all concentrations tested in LPS‐stimulated IPEC‐J2 cells, suggesting that the extracts may attenuate LPS‐induced inflammatory signaling through modulation of the TLR4 pathway. Although the present study did not investigate the precise molecular mechanism, several mechanisms may explain these observations. Polyphenols have been reported to interfere with the TLR4 pathway either by limiting LPS–TLR4 complex formation at the cell surface or by suppressing downstream intracellular signaling cascades. In particular, inhibition of the MyD88‐dependent pathway has been associated with reduced activation of NF‐κB and MAPK signaling, resulting in decreased transcription of pro‐inflammatory mediators such as IL‐6 and IL‐8. Therefore, the observed downregulation of TLR4 expression may reflect reduced receptor activation and/or suppression of downstream signaling rather than direct competitive inhibition of LPS binding. Further studies investigating adaptor proteins such as MyD88, TRIF, and downstream NF‐κB activation are required to elucidate the precise molecular targets of rooibos polyphenols. These findings are supported by the observations reported by Pringle et al. (2018).

The intestinal barrier is characterized by its selective permeability, which is regulated by tight junction proteins. These proteins contribute to barrier‐protective functions by preventing the passage of harmful substances from the gut lumen across the barrier and into the bloodstream. Tight junction proteins, including Claudin, Occludin, and Zonula, provide structural support to maintain the barrier integrity (Citi et al. 2024; Dmytriv et al. 2024). Under inflammatory conditions, pro‐inflammatory cytokines have been reported to cause disruption, relocation, structural changes, and alterations in gene and protein expression of the tight junctions (Contreras‐Ruiz et al. 2012). In this study, Zonula Occludens‐1 (ZO‐1) was selected as a marker to investigate the barrier protective effect of ethanolic rooibos. ZO‐1 is a connected protein, linking the transmembrane tight junction (Claudin and Occludin) with the cytoplasmic intracellular cytoskeleton through F‐actin, providing a stability for tight junctions (Fanning et al. 1998) In this study, the mRNA expression levels of the tight junction proteins ZO‐1, Occludin, and Claudin‐4 were assessed to evaluate epithelial barrier integrity. Dexamethasone treatment increased their expression, demonstrating its protective effect on epithelial junctional complexes. Both unfermented and fermented rooibos extracts improved the expression of these tight junction markers suggesting a role in barrier restoration. Notably, the unfermented extract showed higher induction of *ZO‐1* and *Occludin* gene expression, while the fermented extract markedly enhanced *Claudin‐4* expression at 0.1 mg/mL, exceeding dexamethasone levels. Collectively, these findings indicate that both the rooibos extracts can counteract LPS‐induced epithelial disruption by upregulating tight junction‐associated genes, thereby supporting intestinal barrier repair and protection under inflammatory conditions. These gene‐expression responses did not consistently demonstrate a monotonic concentration‐dependent pattern across all genes and extract types. Rather, the effects were gene‐ and extract‐specific, with clear concentration‐related responses observed for some endpoints, while others displayed non‐linear patterns. The non‐linear responses observed for some genes may be related to the complex phytochemical composition of rooibos extracts, which contain multiple bioactive constituents that can influence different cellular signaling pathways. Interactions among constituents within complex botanical extracts may contribute to concentration‐dependent differences in biological responses (Caesar and Cech 2019; Zhou et al. 2016).

The transepithelial electrical resistance (TEER) results provide additional functional confirmation of the protective effects of rooibos extracts on epithelial barrier integrity. LPS exposure significantly reduced TEER values compared to the untreated control, indicating compromised tight junction function and increased paracellular permeability in inflamed IPEC‐J2 monolayers. Both unfermented and fermented rooibos extracts improved TEER, although their responses differed slightly. The unfermented extract at 0.5 mg/mL and fermented extract at 0.1 and 0.05 mg/mL significantly increased TEER values, suggesting effective protection of barrier integrity. The fermented extract's increased performance at lower concentrations implies enhanced bioactivity or better cellular uptake due to fermentation‐associated phytochemical modifications. Together with the gene expression data for *ZO‐1*, *Occludin*, and *Claudin‐4*, these findings demonstrate that rooibos extracts can preserve epithelial tight junction structure and function under inflammatory stress, contributing to overall barrier protection. The fluorescence intensity of ZO‐1 confirmed that the unfermented tea at 0.5 mg/mL showed the best protection and reflects epithelial barrier integrity and aligns closely with the TEER findings.

Fermentation/oxidative processing of rooibos induces a qualitative shift in the phenolic profile, converting abundant dihydrochalcones such as aspalathin into flavanone C‐glucoside isomers (notably (S)‐ and (R)‐eriodictyol‐6/8‐C‐glucosides) and altering the relative abundance of flavone C‐glycosides (e.g., orientin, isoorientin) (De Beer et al. 2019; Marais et al. 2000). These structurally modified metabolites may possess distinct physicochemical and redox properties compared with the parent compounds and thereby display different potencies and molecular targets. Therefore, a lower mass concentration of the fermented extract can produce comparable inhibition of inflammatory signaling and preservation of tight‐junction function to that observed with higher concentrations of the unfermented extract. At the mechanistic level, flavonoid derivatives such as eriodictyol and related metabolites can inhibit NF‐κB signaling and/or activate the Nrf2 cytoprotective mechanism, that are linked to reduced proinflammatory cytokine production and maintenance of tight‐junction proteins (e.g., ZO‐1, Occludin, Claudin‐4) in epithelial models (Lee et al. 2015; Suzuki and Hara 2009). Importantly, many polyphenols and their oxidation products exhibit a hormetic dose–response where at low‐to‐moderate concentrations they act as signaling modulators and promote adaptive antioxidant responses, whereas at higher concentrations redox‐active metabolites may become pro‐oxidant, generate reactive oxygen species or electrophilic intermediates (quinones) that impair mitochondrial function (ATP collapse) and cause protein damage leading to cytotoxicity (Forman et al. 2014; Mattson 2008; Skaperda et al. 2021). This combination of compositional alterations likely modifies membrane interactions, molecular target engagement, and the dose‐dependent redox behavior of the enriched fermented rooibos extract, thereby providing a plausible explanation for the comparable protective effects of the fermented rooibos extract at low concentrations and its increased cytotoxicity relative to the unfermented extract at higher concentrations.

More importantly, the increased polyphenol content was accompanied by biological responses that differed from those observed with aqueous extracts. Whereas the previous aqueous study demonstrated that fermented rooibos exhibited the strongest anti‐inflammatory activity while unfermented rooibos primarily preserved epithelial barrier integrity (De Bruyn‐Orr et al. 2025), the enriched ethanolic extracts displayed distinct concentration‐dependent effects. The unfermented ethanolic extract retained superior antioxidant capacity and exhibited enhanced suppression of inflammatory mediators together with improved restoration of epithelial barrier integrity. Conversely, the fermented ethanolic extract remained biologically active despite its lower total polyphenol content, achieving anti‐inflammatory and barrier‐protective effects at substantially lower concentrations, while simultaneously exhibiting increased cytotoxicity at higher doses. These findings indicate that biological activity is determined not only by total polyphenol abundance but also by the qualitative changes in flavonoid composition introduced through both fermentation and solvent extraction.

Collectively, the aqueous and ethanolic studies should therefore be viewed as differentially complementary rather than being regarded as similar extraction methods. The aqueous extracts represent the traditional dietary preparation consumed as rooibos tea, whereas the ethanolic XAD‐enriched extracts represent concentrated phytochemical preparations more relevant to the development of functional ingredients, nutraceuticals and phytopharmaceutical products. By directly comparing these chemically distinct extracts, the present study demonstrates that extraction methodology is a critical determinant of phytochemical composition, dose–response behavior and biological efficacy. These findings provide new insight into how extraction processes can be used to optimize rooibos bioactivity for specific therapeutic applications rather than simply increasing polyphenol yield. Studies have shown the absorption and bioavailability of phenolic compounds in the gut; however, further studies are warranted when introducing enriched extraction techniques (Kreuz et al. 2008; Muller et al. 2021). The use of a phytosome/phenolipid formulation could enhance the bioavailability of an enriched extract, thereby optimizing delivery (Human et al. 2023). The current study demonstrates the use of specific extraction as a means to optimize and refine rooibos for a potential therapeutic product and promotion of health.

## 5 Conclusion

Overall, this study showed that both rooibos enriched ethanolic extracts have significant antioxidant, anti‐inflammatory, and barrier protective properties in an LPS‐induced inflammatory model. The unfermented and fermented extracts demonstrated different bioactivity due to the difference in phenolic content. The unfermented ethanolic extract, characterized by its high phenolic content, particularly aspalathin, showed to have superior antioxidant capacity and demonstrated protective responses at higher extract concentrations. On the contrary, the fermented extract, due to the modified phytochemical profile, displayed bioactivity and downregulation of mediators at higher concentrations. Both extracts maintained the intestinal barrier integrity, as shown by the increased expression in tight junction genes (ZO‐1, Occludin, and Claudin‐4). This was further substantiated by increased TEER values. When comparing the extracts, the fermented extract had a lesser therapeutic effect, and this could be attributed to the fermentation‐derived effects. The findings of this study emphasize the importance of extraction methods, extract processing, and dose in determining the efficacy of rooibos polyphenols. Furthermore, both unfermented and fermented rooibos may offer protection against intestinal inflammation. The unfermented providing strong antioxidant properties and the fermented low‐dose anti‐inflammatory activity. Future studies should investigate the molecular mechanisms underlying the anti‐inflammatory effects of rooibos extracts, including the TLR4/MyD88/NF‐κB signaling pathway, evaluate their efficacy in relevant in vivo models, and assess the stability and bio‐accessibility of these ethanolic extracts during simulated gastrointestinal digestion to further support their development as potential nutraceuticals.

## Author Contributions

**Mariam Rado:** data curation, formal analysis, investigation, methodology, validation, visualization, writing – original draft, writing – review and editing. **Stefan Abel:** conceptualization, methodology, resources, software, supervision, validation, visualization, writing – review and editing. **Claudia Abrahams:** data curation, formal analysis, investigation, methodology, writing – original draft, writing – review and editing, validation, visualization. **Shana De Bruyn‐Orr:** conceptualization, methodology, formal analysis, data curation, investigation, project administration, software, validation, visualization, writing – original draft, writing – review and editing. **Mariska Lilly:** conceptualization, formal analysis, funding acquisition, investigation, methodology, project administration, resources, software, supervision, validation, visualization, writing – review and editing, writing – original draft.

## Funding

This work has been supported in part by the South African Rooibos Council (SARC) and additionally, we acknowledge the National Research Foundation (NRF) for providing a Postgraduate bursary to support this study.

## Ethics Statement

The authors have nothing to report.

## Consent

The authors have nothing to report.

## Conflicts of Interest

The authors declare no conflicts of interest.

## Data Availability

All obtained data have been included in the manuscript.
